# Minor covid-19 association with crime in Sweden

**DOI:** 10.1186/s40163-020-00128-3

**Published:** 2020-10-11

**Authors:** Manne Gerell, Johan Kardell, Johanna Kindgren

**Affiliations:** 1 Department of Criminology, Malmö University, Malmö, Sweden; 2 Intelligence Unit, National Operations Department, National Police of Sweden, Stockholm, Sweden

**Keywords:** Corona, Covid-19, Crime trends, Police reported crime

## Abstract

The covid-19 disease has a large impact on life across the globe, and this could potentially include impacts on crime. The present study describes how crime has changed in Sweden during ten weeks after the government started to implement interventions to reduce spread of the disease. Sweden has undertaken smaller interventions than many other countries and is therefore a particularly interesting case to study. The first major interventions in Sweden were implemented in the end of week 11 (March 12th) in the year 2020, and we analyze police reported crimes through week 21 (ending May 24th). Descriptive statistics are provided relative to expected levels with 95% confidence intervals for eight crime types. We find that total crime, assaults, pickpocketing and burglary have decreased significantly, personal robberies and narcotics crime are unchanged. Vandalism possibly increased somewhat but is hard to draw any firm conclusions on. The reductions are fairly small for most crime types, in the 5–20% range, with pickpocketing being the biggest exception noting a 59% drop relative to expected levels.

## Introduction

With a new corona virus spreading rapidly around the world during 2020 it has a tremendous impact on many countries, both directly through the disease it causes, covid-19, and indirectly through the measures governments and other actors take to combat its spread. It appears very likely that it will have a large impact on crime, but differently so for different types of crime (Eisner and Nivette 2020). In this paper we will outline how different types of crime has changed in Sweden since March 12th when the first major intervention against covid-19 was implemented and through the following ten weeks. Sweden has taken much smaller measures to combat the disease than many other countries and has not instituted a lock-down nor compulsory closing of schools. Most Swedish interventions are recommendations to the population on behavioral change to promote social distancing. This sets Sweden apart from many other countries which have affected stricter measures to combat covid-19. The present paper therefore provides a case study of the covid-19 association with crime in a country with less strict measures taken to combat the virus. The impacts on crime in Sweden nevertheless appear to be significant from the onset of major interventions against covid-19 on March 12 through the following ten weeks. We consider changes in total reported crime, outdoors assault, indoors assault, personal robberies, residential burglary, commercial burglary, narcotics crime, pickpocketing and vandalism. We show that most crime types have experienced significant, but fairly small decreases after the government implemented interventions to combat the spread of covid-19.^1^

### Literature review

There has already surfaced many media-reports, and some quick academic analyses on the effect of the covid-19 virus on crime. As noted by Eisner and Nivette (2020) the biggest impact of the virus on crime is likely to come from measures to combat the virus that promotes social distancing. They hypothesize that street violence will decline as people stay at home, while domestic violence will increase. In addition, they hypothesize several other potential mechanisms, for instance that child maltreatment may increase as anxiety rises due to the virus and lockdown and that alcohol sales bans in parts of the world can lead to major declines in violence.

There are a few published studies, and several pre-prints dealing with this issue empirically. Matt Ashby (2020a) has published a paper considering changes to reported crime showing the impact on crime in 16 US cities. Ashby finds no impact on crime before march, and notably that there are large differences between cities. Most of the changes to crime in his models were non-significant using a 99% confidence interval, but a few significant results were found. One city saw significant increases in indoors assault, three cities saw decreases of residential burglary, one city for non-residential burglary and five cities for theft from motor vehicles. While theft *from* motor vehicles thus tended to decrease, theft *of* motor vehicle saw very diverging results, with two cities seeing significant decreases, and three increases (Ashby 2020a). In a follow up study looking at calls for service Ashby (2020b) similarly finds that there was a delay after corona hit ten US cities before calls to service started to decline. For crime related calls for service he concluded that while some cities saw decreases, the over all pattern was that more than half the cities studied had levels of calls for service that were of an expected level for each crime type studied.

Campedelli et al. (2020) analyzed crime in Los Angeles until March 28th, and found no change to homicide, assault with deadly weapon, intimate partner violence, vehicle theft or burglary. They did however note a small reduction of assaults, and somewhat larger reductions in shoplifting, theft and robberies as well as for total crime (Campedelli et al. 2020). Mohler et al. (2020) also studied Los Angeles, in addition to Indianapolis. They note that the strongest impact appears to have been that traffic stops decreased, while calls for service for domestic violence increased. The increase in calls for domestic violence however did not manifest itself in elevated levels of aggravated assaults. The findings on intimate partner violence were somewhat similar in a study by Piquero et al. (2020) on domestic violence in Dallas. They noted a short-term spike after lockdown, but after a couple of weeks levels of domestic violence resumed to normal.

In Australia meanwhile, Payne and Morgan (2020a, b) found that for March 2020 assaults, serious assaults, sexual violence and breaches of domestic violence orders were not significantly different from the forecast. They note however that for serious assaults and sexual violence the outcome was near the lower end of the confidence interval, and caution against too early conclusions as the social distancing interventions were introduced in the second half of the month (Payne and Morgan 2020a). Payne et al (2020) studied violence in Queensland, and found that simple assault, serious assault, sexual offences and breaches of domestic violence orders all fell in March and April of 2020, but that the drop only was significant for serious assault and sexual offences in April. In a third paper, Payne and Morgan (2020b) consider how property crimes have changed during the covid-19 time period. They found that shop theft, other theft and credit card fraud dropped significantly, while property damage, burglary and motor-vehicle theft was non-significantly affected.

Halford et al. (2020) studied how covid-19 affected mobility and crime in the UK and found that all crime types but motor-vehicle theft dropped. Theft and shoplifting saw the biggest decreases, and notably declines began before a lockdown was instituted. They also found that weekday effects that normally can be seen in the data appeared to be more absent during the covid-19 period, with no decreases in shoplifting on Sundays and less prominent increases of assault on weekends. The changes were tied to changes in mobility at different types of locations. The reduction in shoplifting was for instance strongly associated with less mobility in retail areas, whereas the reduction in burglary was associated with more mobility in residential areas.

Taken together these findings show that crime has changed, but differently for different crime categories and in different geographical contexts. These studies also highlight the methodological preferences among scholars interested in the topic of covid-19 and crime, with several opting to use forecasting models such as seasonal auto-regressive integrated moving average (SARIMA) (see Payne et al 2020 and Ashby 2020a for discussion of the technique), and others using interrupted time series (Hodgkinson and Andresen 2020) or Bayesian structural time-series (BSTS) (see Campedelli et al 2020 for a discussion).

While more academic research on the effect of covid-19 will be forthcoming, there is a substantial body of research on the impact of other types of crisis on crime, notably in the form of hurricanes in the US. One study on the relationship of disasters with crime in Florida found that natural disasters were associated with reductions in crime overall, but increases in relationship violence (Zahran et al. 2009). This is in line with the routine activities perspective which suggests that the congruence of potential offenders, suitable targets/victims and absence of capable guardians can explain many crime trends (Cohen and Felson 1979). Exceptional events such as a disaster can have a big impact on the lifestyles and routines of people and thus affect such congruences (Andresen and Tong 2012).

If a disaster results in more people staying at home, there will be more guardianship of homes which should reduce crimes such as burglary. There will also be less people out and about, so fewer potential targets for crimes such as pickpocketing or outdoor violence. There will however be more domestic violence offenders spending time with their partners as potential victims. The type of crisis and the measures taken to combat it will however also have a big impact, which is underscored by other studies on the topic. A study of the hurricane Katrina for instance found that residential burglary increased, which is linked to the fact that people were told to evacuate their homes (Leitner and Guo 2013). This, again, is consistent with changes to routine activities. With fewer people in their homes, there are more homes with lacking guardianship which should result in more burglaries. A similar finding was noted in relation to the hurricane Hugo, which was associated with large changes in people’s daily lives and with increases in reported burglary and people carrying weapons (LeBeau 2002). All of this is in line with the hypothesis by Eisner and Nivette (2020) that lockdowns and other efforts to combat the virus will have a big effect on crime through how it changes routine activities, and through how it affects anxiety and other moods.

It should however be noted that a meta-study on violence against children in the context of disasters found no clear evidence that there was more, nor more severe, violence against children during crisis (Cerna-Turoff et al. 2019). This underscores the uncertainty on many of the issues at hand, and the need for more empirical evidence on how crime changes during a major crisis such as the covid-19 outbreak currently ravaging the world.

### The Swedish context

While Sweden has taken many measures to combat the covid-19 outbreak, other countries have done even more. This means that the association of covid-19 measures with crimes in Sweden is likely to be weaker than in other countries. Below we will outline how Sweden has responded to the outbreak. As is shown in Table 1, the first major intervention impacting on public life in Sweden took place on March 12th, which was in the end of week 11. We therefore expect that an impact from the virus on crime should start to materialize from week 12. The police estimate that some crimes are heavily affected by efforts in other countries as well, mainly through impacting the travels of international theft groups. Most limits on border crossings and similar were however around the same time, with week 11 marking the start of interventions across Europe. The main interventions are presented in Table 1 below.

**Table 1:** Major interventions against the covid19 outbreak in Sweden

Week (date)	Intervention
11 (12/3)	Gatherings of > 500 people banned (Folkhälsomyndigheten 2020a)
11 (13/3)	Anyone with cold symptoms asked to stay home (Folkhälsomyndigheten 2020b)
12 (16/3)	Anyone over age 70 asked to minimize physical contact (Folkhälsomyndigheten 2020c)
12 (17/3)	High schools and universities to teach from distance (Folkhälsomyndigheten 2020d)
12 (19/3)	Anyone asked to refrain from unnecessary travels (Folkhälsomyndigheten 2020e)
13 (24/3)	Restaurant, café, bar mandated to limit crowding (Folkhälsomyndigheten 2020f)
13 (27/3)	Gatherings of > 50 people banned (Folkhälsomyndigheten 2020g)
14 (1/4)	General recommendations to businesses and associations to limit social interaction. Employers recommended to encourage work from home. Public transport to reduce crowding (Folkhälsomyndigheten 2020h)
18 (29/4)	Pregnant women with health problems recommended to limit social contacts as much as possible (Folkhälsomyndigheten 2020i)

The worst affected region in Sweden, Stockholm region, has released data on the use of public transport. It shows a small reduction in week 9–10, and a large drop beginning in week 11 which then stabilizes at a level of about 35–40% of normal public transport usage (SLL 2020). This suggests that some social distancing took place in early March, but that the biggest impact will be seen in the second half of the month.

## Methods

In this paper we estimate whether crime in week 12 to week 21 differ from what we’d expect in relation to both prior crime levels in 2020 and weekly trends in prior years.

The analysis for this paper uses the standard Swedish police procedure to track and project crime trends. It is based on a basic method devised by the National Council for Crime Prevention to track crime trends at a local level (BRÅ 2001). Monthly crime projections are simply inferred from prior years of crime, using the median to reduce the impact of outliers. The method is based on the idea that crimes roughly follow a poisson distribution,^2^ and so 95% confidence intervals (CI) are calculated by the formula ± 1.96*SQRT(n). The method is meant to be used on monthly rates, but here we opt to track weekly changes instead. For large numbers of crimes, the confidence intervals become very small, and not very meaningful,^3^ but for specific crime types it gives a reasonable estimate as to what can be considered natural fluctuations in crime and what constitutes a meaningful change. This is a less robust method of forecasting than what many other studies have used. It was used since it was the preferred method to use by the Swedish police intelligence unit, and an advantage of using it is its simplicity and non-technical nature which makes it more available for use by practitioners.

**Fig. 1:**
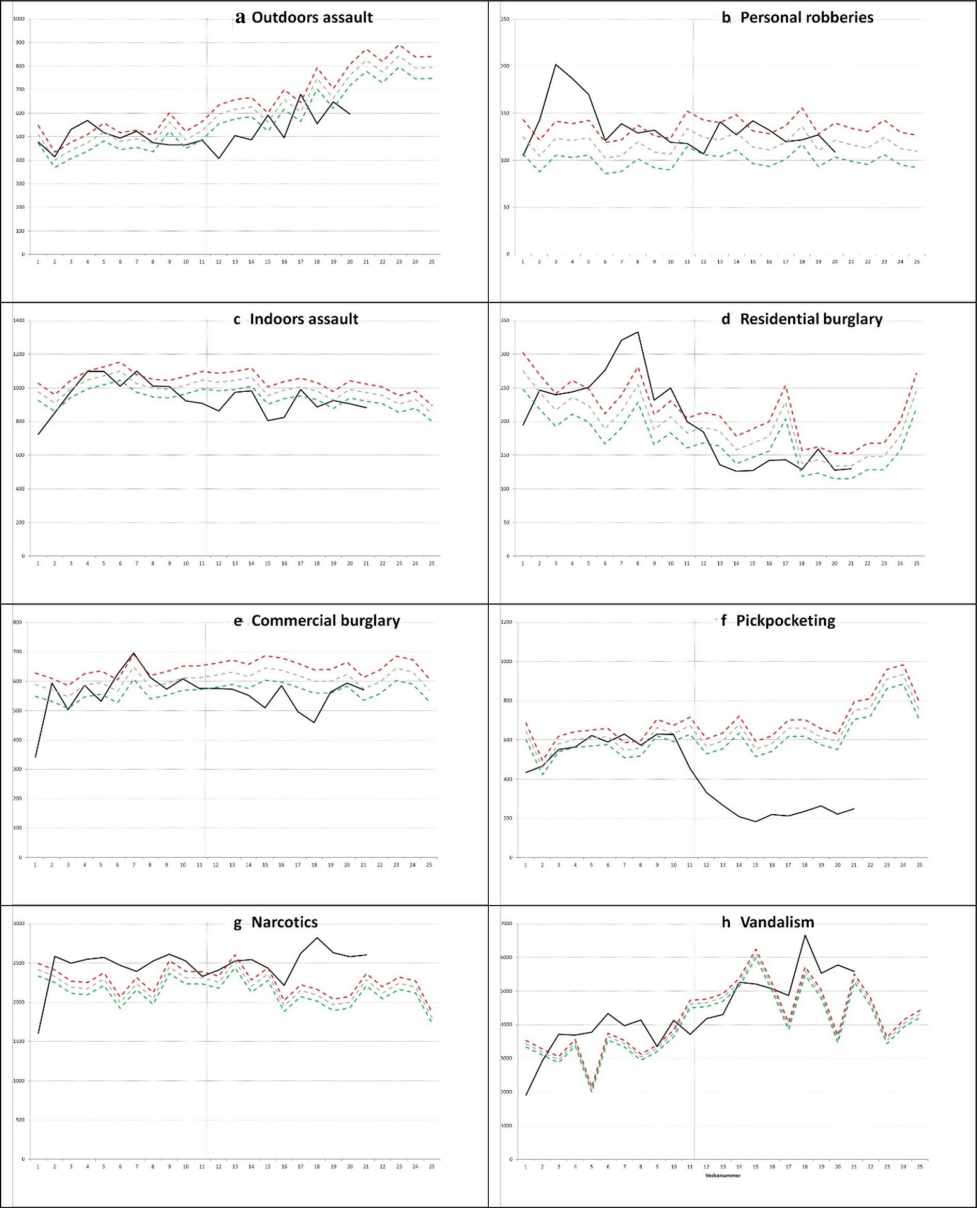
Projected and actual crime rates for 8 crime types with 95% confidence intervals (lower = Min, upper = Max). First row left **a** Outdoors assault; First row right, **b** Personal robberies; Second row left, **c** Indoors assault; Right **d** Residential burglary; Third row left **e** Commercial burglaries; Third row right **f** Pickpocketing; Last row left **g** Narcotics; Last row right **h** Vandalism

### Research design

As the method employed takes no account of whether there are yearly trends, we adjust our analysis somewhat. To test if there appears to be a yearly trend, we check whether 2019 diverge from the median of the three prior years using the same method outlined above. If 2019 differs, we use the 2019 data as our main comparison rather than the median.^4^ In the next step we compare the change in crime nine weeks before (week 3–11) and after (week 12–20) covid19-interventions for 2020 in relation to the 2019 level or the median for 2016–2019. If the change is similar it does not appear to be a covid19 association with crime. If, however, there is a difference, we then compare the difference to the confidence interval to see if appears to be significant, and if so, how large it is.

### Data

This paper focuses on describing the crime trends associated with the covid-19 outbreak for total crime, and eight crime categories:

- Outdoors assault
- Personal robberies
- Indoors assault
- Residential burglary
- Non-residential burglary
- Pickpocketing
- Narcotics crime
- Vandalism

All data is taken from the official Swedish police data base over reported crimes, with the data pulled out on Wednesday the 27th of May, including data recorded up to Sunday 24th of May (Table 2, Additional files 1 and 2).

**Table 2:** Descriptive statistics for crimes in the paper, covering week 1–25 for 2016–2019, and week 1–21 for 2020

Crime type	N	Weekly mean	Standard deviation
Total crime	44,19,614	36,526	2811
Outdoors assault	71,825	594	137
Indoors assault	1,18,541	980	89
Personal robberies	14,637	121	22
Residential burglary	29,903	247	71
Commercial burglary	72,018	595	65
Pickpocketing	87,964	727	222
Narcotics	2,54.895	2107	285
Vandalism	482,613	3989	1110

Total crime is the total weekly inflow of reported crimes. Violence is measured with three indicators, assault in public environments, indoors assault^5^ and personal robberies. It should be noted that in Swedish law, assault is by definition a physical assault, typically a blow with a hand or foot. Residential burglary is measured using completed burglaries in both small houses and apartments. Non-residential burglary consists of burglary of all facilities that are not homes, basements, attics or a holiday home.^6^ Narcotics crime consists of all narcotics crime, but the bulk of it is made up of possession or use. Vandalism comprise several crime codes of vandalism, graffiti and similar.^7^ Pickpocketing is measured with two crime codes specifically designed for such theft.

For this study we use crime data until week 21 which is shown in Fig. 1. In our numerical analysis we however only include week 12–20 as we expect the crime data for week 21 to be adjusted upwards due to the lag in data registration which is discussed below.

### Lag in data registration impact

We use the date of registration, rather than the date when the crime is committed, to reduce problems of crimes being recorded retrospectively as we want to track crime in almost real time. There is however a risk that crimes get re-coded into different crime categories or that crimes are registered later than the date they it is supposed to have been recorded. To estimate the effect of lags in data registration we coded all crimes during week 13, and then redid the analysis for week 10, 11 and 12 during week 14 to see how much it changed. The week 12 total crime count was then adjusted upwards by 1.1%, the week 11 count by 0.3% and the week 10 count by 0.04%. This means that we can generally expect our crime counts to be a slight underrepresentation for the latest two weeks included in the analysis, and we will consider this in our discussion of how the changes in crime can be interpreted, and do not emphasize crimes of the last week with available data, week 21.

## Results

Total crime is down significantly, both compared to prior levels during the year^8^ and compared to the projection based on previous years. In the beginning of the year crime was higher than expected, but it has since been trending down continuously. Week 12 to 20 in 2020 registered 6.5% less crimes (n = 21 518) than week 6 to 11 (2019 − 5.3%, median − 2.9%). From week 12 there is a notable drop in the number of reported crimes. In the following weeks this appears to have stabilized at a lower level, but not dropping further relative to expected levels, in spite of further interventions to combat covid-19 being introduced. Relative to the projected rate the drop is in total 9.4%, or 30 980 fewer crimes, in week 12 to 20.

The police divide police reported crimes into 14 main categories. 12 of these consist different types of crime, one consists of incidents that are deemed not to be a crime, and one consists of incidents without a category, but that is very minor with just 0.006% of the total. For the other 13 categories there is a reduction in reports between week 11 and week 13 in 11 categories. In absolute numbers the biggest drops are for the “not a crime” category (− 987) and the “theft, not in a shop” category (− 953). Relatively speaking the biggest drop was for “economic crimes” (− 50%), but this category is very rare, with just 3 crimes reported in week 13. For more common categories the biggest drops are for “not a crime” (− 24%), “theft from a shop” (− 23%), “local traffic codes” (− 15%), and “theft, not in a shop” (− 14%). Two categories saw increases, “vandalism” (+ 556, 14.7%) and “narcotics” (+ 151, 6.5%).

Below we will now look more closely on the specific crime categories of interest in the present study.

### Violence

Outdoors assault dropped in week 12, and has since increased, but less so than expected given prior years and the seasonal effect on assaults when spring comes. Week 12 to 20 in 2020 was 9.7% higher than week 3 to 11 (2019: + 36.9%, median + 31.0%). Compared to the median the 2020 rate was significantly lower in seven of the nine weeks post-covid-19. This corresponds to a relative reduction of about 20.8%, or 105 fewer assaults per week (Fig. 1a).

For personal robberies week 12 to 20 was − 14% compared to week 3 to 11 (2019: + 7.9%, median + 4.3%). Most of this change was however due to an unusual spike in personal robberies in week 3 to 5, and after that the robberies have tended to be at an expected level. There does not appear to be any change associated with covid19 interventions (Fig. 1b).

Indoors violence dropped during week 9 to 11, and since appear to have stabilized at a level that is slightly lower than expected. Week 12 to 20 was − 10.6% compared to week 3 to 11 (2019: + 1.7%, median − 3.3%). The relative change post-covid-19 compared to the median level is -7.3%, corresponding to 74 fewer indoors assaults per week (Fig. 1c). As mentioned above crime fell before covid19 had a major impact on Sweden, however.

### Theft

For residential burglary, the rate was significantly lower in 2019 which thus is used in Fig. 1d. Week 12 to 20 in 2020 had − 45.6% compared to week 3 to 11 (2019: − 20.3%, median − 28.8%). This difference is however partly driven by higher than usual rates in the beginning of the year, and a decrease that began before covid-19 (week 9 saw a big drop). Burglaries have registered lower than expected levels for five of the nine weeks after major interventions started to be implemented. The relative difference post-covid-19 compared to 2019 is − 29.1%, or 76 fewer burglaries per week.

Commercial burglary dropped significantly below the expected level in week 12, but just barely. It has been below expected levels six of the nine weeks measured. Week 12 to 20 in 2020 was -7.6% compared to week 3 to 11 (2019: + 2.4%, median + 4.8%). This corresponds to a 12.4% decrease post-covid-19, or 73 fewer commercial burglaries per week (Fig. 1e).

Pickpocketing had a significantly lower rate in 2019, and the 2019 comparison is therefore used in Fig. 1f. The 2020 rate was very similar to 2019 up until week 10, and since has dropped fast. Week 12 to 20 had − 59.0% compared to week 3 to 11 (2019: + 0.3%, median + 4.4%). This corresponds to a − 59.4% change post-covid-19, or 346 fewer crimes per week.

### Other crimes

Narcotics crime is mostly a measurement of police activity. It has been at a high level in 2020, and was significantly higher in 2019 as well, which is thus used in Fig. 1g. Week 12 to 20 was + 1.4% in 2020 compared to week 3 to 11 (2019: + 2.5%, median + 3.7%). There does not appear to be any general change post-covid-19, although the last few weeks included do have elevated levels.

Vandalism is the final type of crime we track. Vandalism was significantly higher in 2019, which is therefore used in Fig. 1h. Week 12 to 20 in 2020 was + 34% compared to week 3 to 11 (2019: + 45.3%, median + 13.9%). Using the 2019 data there is therefore a decrease post-covid-19 of 4.9%.

## Discussion

In the present paper we have presented weekly crime counts for eight crime categories to try to discern whether the covid-19 outbreak and interventions to combat it are associated with any short-term changes to crime. Our findings suggest that total crime is down significantly, as are most of the crime categories studied. Both outdoors assault and indoors assault are down, as are residential and commercial burglary. Pickpocketing sees the biggest decreases. There is no discernible association with rates of personal robberies or narcotics crime. Vandalism is registered for a slight increase compared to 2019, but a decrease compared with the median value for prior years. In addition, rates of vandalism are largely (~ 50%) driven by Stockholm county, and spikes can be caused by the public transport company mass reporting graffiti and similar when they find the time to do so, which means it is quite volatile. We therefore consider changes to vandalism to be non-conclusive.

All three theft-related crimes saw significant drops post-covid-19. The drop in residential burglaries could be associated with an effect of more people being at home, thus increasing guardianship (Cohen and Felson 1979; Halford et al 2020), but possibly there could also be an effect from fewer international crime groups being around due to border closings around Europe. The latter mechanism could affect commercial burglaries as well. It is possible that other countries undertook interventions earlier than Sweden, which could explain why the drop in burglary began before the Swedish government intervened substantially. An alternative explanation is that parts of the population may have practiced voluntary social distancing which could have an effect. Social distancing, and possibly border closing, are likely to have affected pickpocketing as well. With less crowded public transport and fewer people outdoors, this is a type of crime that should become harder to commit. Halford et al. (2020) showed that non-shoplifting theft was reduced in relation to lower mobility in retail areas, workplaces and public transport which fits with this idea. Either way, there is a substantial drop coming after the Swedish covid-19 interventions that likely, at least in part, is attributable to the interventions against the virus. Some of these findings are consistent with what Payne and Morgan (2020b) found regarding property crime in Australia, while some are not. They did not identify any significant drops in burglary, but did find other types of theft was reduced, including a crime category that appear to capture pickpocketing. This, again, highlights the differential association of covid-19 and crime across different crime types and geographical contexts.

The fact that both outdoors and indoors assault have decreased is somewhat surprising. Reductions in outdoors assault were to be expected due to fewer people being out and about, and fewer people in the night life (Eisner and Nivette 2020). Previous research in Sweden have also shown that alcohol consumption in restaurants has a 5–10 times stronger effect on reported outdoors assault than alcohol consumption at home. The latter part of consumption had a stronger effect on domestic violence (Lenke 1990). This is a part of our assumption that indoors assault would increase, which is not to be seen so far. It is however possible that the partial lockdown has affected the ability to report crimes such as relationship crime or violence against children. It may for instance be harder to report crimes to the police when you are indoors with the offender the whole time, and in addition some crimes will be reported by schools or similar, which can be reduced when some people are schooling from home. It should also be stressed that indoors assault started dropping before any major interventions against covid19 were implemented, casting a doubt on whether the lower levels registered later are associated with covid19 at all.

The fact that both assaults and burglary shows fairly small decreases is in line with prior studies on the Covid 19 and crime which mostly finds fairly small reductions in crime, and/or reductions in some cities but not in others (Ashby 2020a; Payne and Morgan 2020a, 2020b; Piquero et al. 2020; Mohler et al. 2020). This is interesting to note as the fairly big changes to routine activities, as measured by mobile phone or public transport data, appear to translate into modest reductions in crime. This was studied directly by Halford et al (2020) who found that changes in mobility was strongly related to changes in crime, but differently so for different crime types.

In Sweden however there has been an attempt not to lock the society down, and modest crime reductions can therefore be viewed as being in line with expectations. The exception to this is the crime of pickpocketing which has seen a very large reduction. This is an interesting finding that warrants further examination in the future but is perhaps not surprising as pickpocketing is facilitated by a crowd (Felson and Boba 2010), and crowds should clearly be influenced by social distancing.

A final finding from the crime trends is that there appears to be somewhat of an uptick in crime in the most recent weeks of the study, and that the clearest relative reduction was around week 13 to 17. A similar phenomenon was noted by Piquero et al. (2020) who found that domestic violence increased, but then returned to normal levels after a few weeks. It is possible that people in general, and criminals in particular, adjust to a new reality and to a new normal, but this remains to be seen in future studies. As we noted in the methods section the last few weeks included in the study are likely to have their crime levels adjusted upwards slightly as well, which may exacerbate this trend. The upwards adjustment however tends to be fairly small for most crime types.

In addition to the above-mentioned limitation the present study uses a very basic method to project expected crime trends. Most papers on this topic have used ARIMA models or similar (Ashby 2020a, b; Payne and Morgan 2020a, b; Piquero et al. 2020) while we opted to use the standard Swedish police method combined with a simple test to see if crime appear to differ in 2019 compared to the previous years to account for yearly trends. We acknowledge that this means our findings are less robust than they would be with an ARIMA model. We do however believe that there is some value in descriptive explorations of crime trends and how they change, and that there may be an advantage in showing how such calculations are done in Sweden and can be done elsewhere with very little technical or statistical knowledge.

## Conclusion

The present study has described crime trends in Sweden in the ten weeks that has passed since the government enacted interventions to combat the covid-19 outbreak. Sweden is a particularly interesting case study for the covid-19 and crime association since it has undertaken less drastic measures to combat the virus than many other countries. With no stay at home orders, and no compulsory closing of most schools or daycares one could expect a weaker association of social interventions and crime in Sweden than elsewhere. The present study does not formally test international differences, and it is difficult to draw any firm conclusions on the topic based on the published findings from other countries so far.

We find that total crime is down significantly, alongside burglary and violence. There is however no association with rates of personal robberies or narcotics crime, and vandalism is difficult to draw any firm conclusions on but does not appear to have seen a major change. Pickpocketing finally has seen a very large decrease. As criminals and law enforcement alike adjust to this new reality, this may well change, and while the last few weeks in the data studied possibly indicates some return to normal, clearer indications of that are yet to be seen. Similarly, future studies should do formal comparisons between countries to attempt quantification of the impact different social distancing interventions can have on crime.

## Supplementary information

**Additional file 1.** Results_week21.

**Additional file 2.** Results_week21_othercrimes.

## Data Availability

The data will be added as supplementary material.

## Footnotes

1 The present article expresses the opinions of the authors and is not the official view of the police authority.

2 Aggregate crime as a poisson (or negative binomial) distribution has been suggested as appropriate for low counts (Osgood 2000). In the present study most crime counts are however fairly large and stable, and thus likely to be more normally distributed. The poisson distribution assumption is however inbuilt in the method employed by the police and therefore used here.

3 Consider for instance the vandalism graph, Fig. 1h. For low volume crimes, such as homicide, the opposite would be true, with confidence intervals becoming very large. In the present paper no such crimes are included.

4 We have employed both methods for all crime types, and in half the cases it makes little difference, but for vandalism, residential burglary, narcotics and pickpocketing there are meaningful differences.

5 Relationship would be of more interest in relation to international research, but the numbers are very volatile. This reflects many changes to crime codes within this crime category, which means we cannot use it to compare with prior years and we therefore opted to use indoors violence as an indirect measurement. All assaults have to be coded as either outdoors/public environment or indoors/non-public environment.

6 Offence codes 0811–0823, 0829.

7 MBK5 is the vandalism group label in the police system.

8 Week 1 only consist of five days in 2020, which means that week 1 tend to have very low levels of crime. This can clearly be seen in Fig. 1, and should be kept in mind when interpreting the Figure.
